# Melanic variation underlies aposematic color variation in two hymenopteran mimicry systems

**DOI:** 10.1371/journal.pone.0182135

**Published:** 2017-07-28

**Authors:** Heather M. Hines, Paige Witkowski, Joseph S. Wilson, Kazumasa Wakamatsu

**Affiliations:** 1 Department of Biology, The Pennsylvania State University, University Park, Pennsylvania, United States of America; 2 Biology Department, Utah State University, Tooele, Utah, United States of America; 3 Department of Chemistry, Fujita Health University School of Health Sciences, Toyoake, Aichi, Japan; University of Akron, UNITED STATES

## Abstract

The stinging hymenopteran velvet ants (Mutillidae) and bumble bees (Apidae: *Bombus* spp.) have both undergone extensive diversification in aposematic color patterns, including yellow-red hues and contrasting dark-light body coloration, as a result of Müllerian mimicry. Understanding the genetic and developmental mechanisms underlying shifts in these mimetic colors requires characterization of their pigmentation. In this study, a combination of solubility, spectrophotometry, and melanin degradation analysis are applied to several color forms and species of these lineages to determine that orange-red colors in both lineages are comprised of primarily dopamine-derived pheomelanins. Until a few recent studies, pheomelanins were thought not to occur in insects. These results support their potential to occur across insects and particularly among the Hymenoptera. Shifts between black and orange-red colors, such as between mimetic color forms of bumble bee *Bombus melanopygus*, are inferred to involve modification of the ratios of dark eumelanins to red pheomelanins, thus implicating the melanin pathway in mimetic diversification. This discovery highlights the need to focus on how pheomelanins are synthesized in the insect melanin pathway and the potential for new pigments to be found even in some of our most well-known insect systems.

## Introduction

Some of Nature’s most astounding diversifications involve Müllerian mimicry. In this form of mimicry, similarly defended organisms within a geographic region converge upon a common warning signal, such as color pattern, that collectively reduce predation. Such frequency-dependent selection has generated geographic mosaics of mimicry, whereby multiple harmful species converge onto a shared color pattern in one region, but shift to converge on another aposematic pattern in neighboring mimicry complexes. The resulting selection landscapes generate species with multiple local color patterns and thus rapid color change at the lineage level. This feature of Müllerian mimicry is implicated in driving color pattern radiations in several systems, such as butterflies [1,2], neotropical frogs [3], vipers [4], and millipedes [5]. Two Müllerian mimicry systems, the velvet ants and bumble bees, are particularly remarkable in the number of species and the diversity and geographic range involved in their mimicry rings.

**Fig 1:**
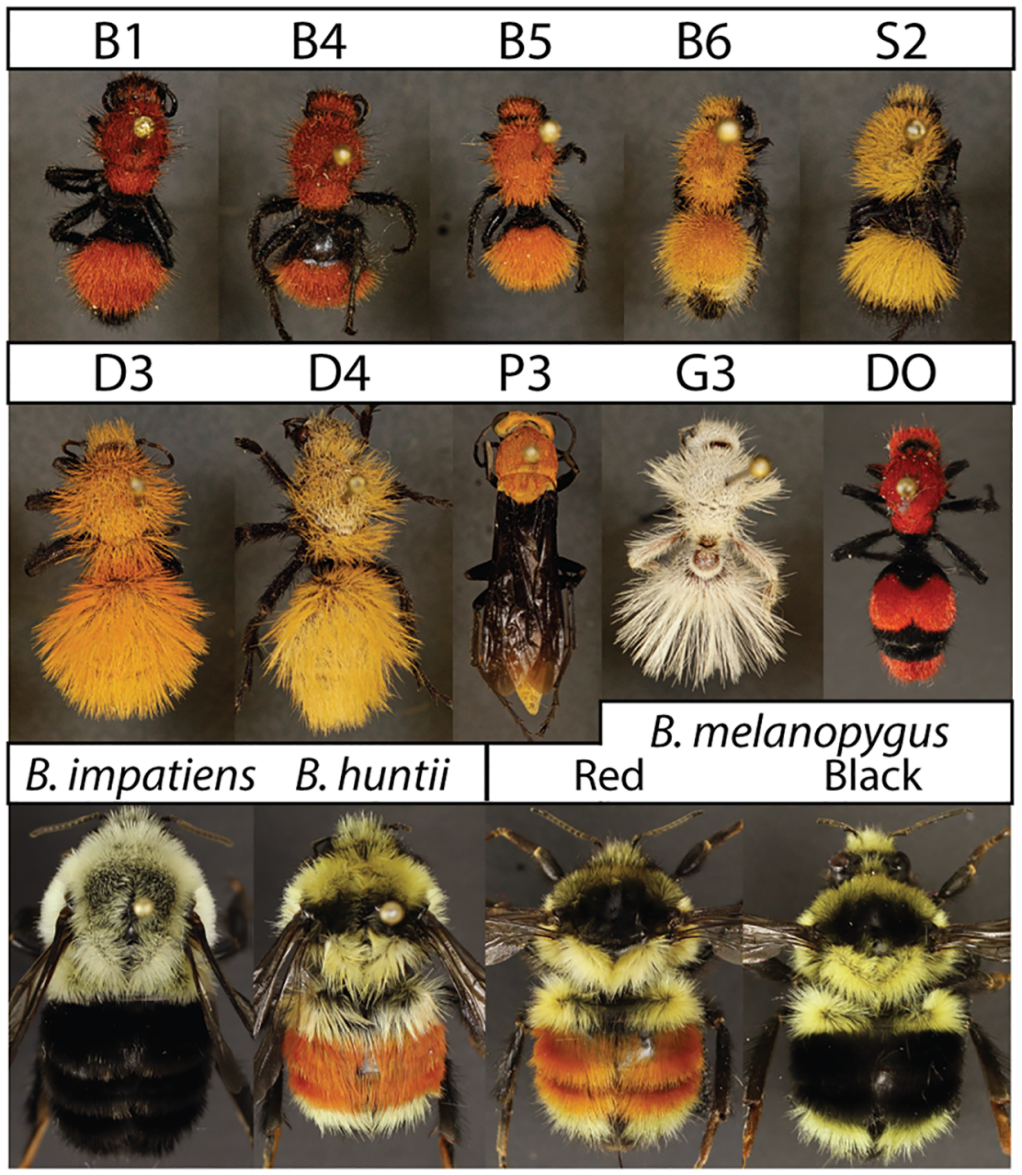
Specimens studied. B1—P3 were used for reflectance and absorbance measurements and represent: **B1** = *Dasymutilla bioculata*, dark orange/red form [USA: Utah: Cache Co., Hyrum; 41.63, 111.86; 7/8/05;T. Bond]; ***B4*** = *Dasymutilla bioculata*, dark orange/red form [USA: Utah: Cache Co., Hyrum; 41.63, 111.86; 8/11/05; T. Bond]; ***B5*** = *Dasymutilla bioculata*, medium orange form [USA:Utah: Emery Co., 25km NNE Hanksville, Gilson Butte Well; 38.58, -110.58; 24.VII.2001; M. Hauser]; ***B6*** = *Dasymutilla bioculata*, light orange form [USA: Utah; Garfield Co.; 37.34, -111.06; 25-July-2001; RW Baumann, RD Gordon, IS Winkler]; ***S2*** = *Dasymutilla scitula* [USA: Utah; Garfield Co., Calf Creek; 37.83, -111.42; VII-30-1982; Griswold/Parkers]; ***G3*** = *Dasymutilla gloriosa* [USA: Utah; Washington Co., Werner Valley, 8mi SE St. George, 37.02533, -113.43408; 25-30.viii.2010; J. Wilson]; ***D3*** = *Dasymutilla satanas*, orange form [USA: Nevada; Rock Valley, Nye Co.; 36.63, -116.31; 7/28/1965; EF Dailey-Attrop]; ***D4*** = *Dasymutilla satanas*, light orange form [USA: Arizona, Parker Dam; 34.30, -114.13; 7-IX-1963]; ***P3*** = *Psorthaspis portiae* [USA: Arizona; Cochise Co., Rte. 666 W. of Wilcox; 32.15, -109.93; 17-July-1991; BP Harris]. Remaining specimens are representatives of species used for degradation analysis as well as absorbance and reflectance measurements. **DO** = *Dasymutilla occidentalis*. All specimens used were female. Specimens were cropped from their original image. DO and the bumble bees were photographed separately from other mutillids and color was standardized between images. Their scaling relative to other specimens is approximate. Specimens in the first row + G3 were increased to 125% of the scale of other specimens to improve visualization.

Velvet ants, wingless stinging hymenopteran wasps in the family Mutillidae, have a widespread global distribution and are recognized for their thick cuticular sclerotization (a type of armor), particularly painful and venomous sting [6–8], and the diversity of color patterns contributed by the setal pile covering their exoskeleton and/or the exoskeleton itself. Coloration in velvet ants occurs in shades of white, yellow, orange-red, and black, and is often similar across much of the body or within major tagma (Fig 1). Although velvet ants worldwide are likely to participate in mimicry, Müllerian mimicry in these wasps has been addressed primarily in New World fauna [9,10]. These studies have revealed geographic convergence in both color pattern and hue of >300 species and ~20 genera onto ~8 New World mimicry complexes. These patterns are also mimicked by sympatric spider wasps [11].

The ~250 species of bumble bees (Hymenoptera: Apidae: *Bombus*) also exhibit high color diversity, with >400 defined color variants, and thus high intraspecific variation. This variation is driven to a large extent by convergence onto >24 generalized mimetic patterns across their Holarctic and South American range [12]. Bumble bee females are venomous through their sting and advertise their toxicity through aposematic coloration of the setal pile that densely coats their body sclerites. Bumble bee colors can vary with each segmental sclerite, with each mimetic pattern having characteristic combinations of shades of yellow, white, orange-red, and black across body segments [13]. While color diversity is primarily attributed to mimicry in these bees, the colors utilized in these regional patterns also are related to climatic factors [12,14].

The diversity of each of these systems informs the evolutionary process and makes these systems especially well-suited for tackling how adaptive diversification happens at a genetic level. Through determining the genetic and developmental processes underlying the color shifts in these two mimetic hymenopteran lineages, we can target hotspots for adaptation, understand how phenotypes arise and sort through time under selection, and how genetic mechanisms enable rapid change. A first step in gaining such insights in these systems is to characterize the end-products of these genetic pathways by identifying the pigments that comprise their aposematic colors. Discovering the pigments will highlight candidate pathways and provide initial insights into the complexity of this regulation. In bumble bees, research [15] has shown that yellow setae are colored by a novel pterin-like pigment and white setae likely lack pigmentation. The red and black colors in the bumble bees were revealed to most likely be melanins, given the solubility of these pigments only in strong heated bases and acids and the characteristic absorbance spectrum (a steadily descending slope from UV wavelengths) of the resulting extracts [15]. Given that red colors typically are not melanins in insects, this result is unusual and thus begs additional validation of pigment chemistry. The nature of the pigments in the velvet ants has not been previously studied.

Across vertebrates two types of melanins are common–the black eumelanins and the red pheomelanins. Yellow-red pheomelanins provide the characteristic colors of red hair in humans and other mammals and red feathers in some birds (e.g., chickens). In insects, melanins are important components of the exoskeleton, not only for the color they provide but for their role in sclerotizing the cuticle and providing immune protection. Until recently, eumelanins alone were thought to be involved in insect melanization, and pheomelanins were thought not to occur in insects and related invertebrates [16]. Discovery of the nature of melanins has been promoted by three primary techniques–using Raman spectroscopy to look for common peak maxima with known melanins [17], by comparison of spectrophotometric absorbance ratios at different wavelengths (650/500nm; [18]), and most precisely, through analysis of breakdown products of melanin extracts [16]. Melanins are particularly challenging to extract, but the nature of these melanins, with regard to whether they are pheomelanins or eumelanins and whether either of these is DOPA or dopamine derived, can be identified by their breakdown products using a refined method [19–21]. In the last two years, two insects have been found to have pheomelanin using these methods. Galván and colleagues [22], using a combination of Raman spectroscopy and degradation product analysis, provide evidence for small amounts of pheomelanins being implicated in the coloration of reddish-brown grasshoppers (*Sphingonotus azurescens*). Subsequently, Raman techniques alone were used [23] to infer the presence of pheomelanins in the brownish leg spots of *Mesopolobus* pteromalid parasitoid wasps.

Our goal is to identify the nature of the pigments in the bumble bee and velvet ant mimetic systems to better understand the mechanisms underlying their mimicry. In velvet ants, we address the unknown nature of the pigments across white, yellow-orange-red, and black color variants. By shifting these colors, these velvet ants are converging onto several different mimicry complexes (e.g., white and yellow variants occur in the North American deserts, more orange-red variants occur throughout the Rockies) thus determining the nature of these variable colors highlights the chemical mechanism of their mimicry. We further refine our understanding of the red and black pigmentation in bumble bees. This involves comparing the nature of melanins in two mimetic forms of the same species, *Bombus melanopygus* Nylander. This species converges onto Pacific coastal vs. Rocky Mountain mimicry complexes by shifting the color of the medial abdominal tergites from black to orange-red, respectively (Fig 1). By comparing the chemistry of setae from these two color morphs, we are able to determine the chemical mechanisms underlying their mimicry. Extracts from additional bumble bees are tested alongside these bees. To determine the nature of these pigments we initially use a combination of solubility, reflectance spectrometry, and absorbance spectrophotometry data. We further refine characterization of inferred melanin pigments in these systems using chemical characterization of melanins through melanin degradation analysis [16]. Our results add to growing evidence of the involvement of pheomelanins in insects and highlight how modifications in the melanin pathway are driving diversification in aposematic mimetic coloration in both of these systems.

## Materials and methods

### Color quantification: Spectrometry & photographic analysis

Color reflectance data was gathered using an Ocean Optics 2000+ Spectrometer (Xenon lamp, 200–850 nm detection, custom 25 micron optic; Dunedin, FL, USA) from white or yellow-red regions of females of 4 different species of velvet ants (Hymenoptera: Mutillidae) including pinned, dried specimens of *Dasymutilla bioculata* (Cresson) (light, medium, and dark/red orange forms), *Dasymutilla satanas* Mickel (both light and medium orange variants), *Dasymutilla scitula* Mickel (light orange), and *Dasymutilla gloriosa* (Saussure) (white); from a female of a spider wasp (Hymenoptera: Pompillidae) species *Psorthaspis portiae* (Rohwer) that mimics the western velvet ants [11]; from the orange-red second and third abdominal tergites of a fresh-frozen queen of the bumble bee *Bombus huntii* Greene (Utah, obtained from J. Strange); and from the black metasomal region of a queen of the bumble bee *Bombus impatiens* Cresson (Fig 1, fresh-frozen from commercial Koppert colonies). Reflectance data were normalized to the same reflectance intensity at 750 nm to allow discrimination of differences in spectral shape.

To accurately assign colors to these specimens for spectral figures we obtained high resolution photographs of pinned specimens using a Canon EOS Rebel T3 camera with 100mm macro lens, a GIGAmacro Magnify2 Robotic Imaging System (Four Chambers Studio LLC, Napa, CA, USA) with Autopano Giga software (Kolor, Francin, France) for stitching, a Zerene Stacker (Zerene Systems LLC, Richland, Washington, USA) to z-stack images, a flash lighting setup that evenly distributes light, and using a color reference standard. Bumble bee and *Dasymutilla occidentalis* (Linnaeus) photographs were obtained separately using a Canon D80 with 50 mm macro lens and a similar lighting set-up. Color data (RGB) was extracted from the main region of colored (white, yellow, or orange-red) setal pile from each specimen in Adobe Photoshop software (San Jose, CA, USA) (abdomens in velvet ants and bumble bees, thoraces in the spider wasp). The central colored area from these specimens was averaged across this region (Filter:Blur:Average) before color determination.

### Pigment class characterization

**Fig 2:**
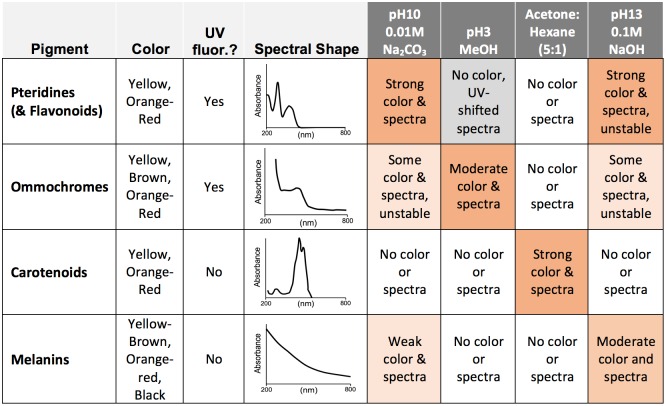
Criteria used for assessing pigment types extracted from insect cuticle based on solubility, UV fluorescence, and spectral shape. While spectral wavelengths shift, general spectral shape is similar across the pigment class. Some deviation from this model could occur given side groups of some members of each class.

We determined the class of pigments involved in coloration in velvet ants and their spider wasp mimics using tests of solubility combined with spectrophotometry. All setal samples were collected by scraping setae off of cuticle with a razor blade. To initially test solubility, we sampled hairs from both a dried female orange variant of *Dasymutilla satanas* (similar to D3, Fig 1) and two fresh-frozen female specimens of *Dasymutilla occidentalis* (red color, DO Fig 1, USA: Alabama, Solon-Dixon Rd.; 31.15, -86.70; viii-viii-2016; D. Owen), and followed a four-step, four-solvent procedure that can distinguish the major pigment classes. Insects typically make yellow-to-red colors using either carotenoids, ommochromes, pteridines, or melanins. These compounds differ both in solubility in different types of solvents but also in the characteristics of their absorption spectra (Fig 2; [24]). To distinguish among these, we ran the setal samples at a ratio of ~1 setae: 2 solvent (v/v) through the following solvents, in order: 1. pH 10 0.1 M Na_2_CO_3_ (visibly extracts pteridine, some ommochrome pigments, small amounts of melanin), 2. pH 3 acidified (HCl) methanol (better extracts ommochromes & extracts pteridine pigments but makes them colorless), 3. acetone:hexane (5:1) mixture (extracts carotenoids), and 4. pH 13 0.1 M NaOH (visibly extracts pteridines and ommochromes but chemical stability is lost over time, and melanins). These samples were processed by vortexing, heating at 80°C for 1 minute, flash cooling on ice for 1 minute, repeating this procedure twice, followed by centrifugation for an additional two minutes. In between samples a filter tube was used to dry the hairs and prepare them for subsequent extractions. The resulting extracts and hairs from each step were inspected for color and extracts were run immediately using 1–2 μl samples on the Nanodrop (Thermo Scientific) spectrophotometer to further determine if any pigment was leached. For the acetone:hexane only visual inspection was used given rapid evaporation of samples during spectrophotometry.

We chose to extract pigments in the 0.1 M NaOH (pH 13) solvent for comparative absorbance measurements across our remaining specimens. This solvent was chosen as it would be able to extract pteridines, melanins, and to some extent ommochromes and thus be most likely to yield a successful extraction, and because this was revealed to be most effective at leaching pigment with the initially tested samples. Extracted pigments with this solvent could be further assessed for pigment class given the shape of the absorbance spectra and fluorescence (Fig 2). This procedure was performed on the same velvet ant and spider wasp samples used for reflectance data (see Color Quantification: Spectrometry & Photographic Analysis). Extraction procedures followed the same procedure outlined above. In some of the resulting spectra there was a slight indication of spectral peaks that did not match melanin chemistry. To determine whether these could be pteridines or ommochromes, we performed extractions on separate female specimens of species yielding extra peaks, namely *D*. *scitula*, *D*. *gloriosa*, and *Psorthaspis portiae*, using pH 10 0.01 M Na_2_CO_3_. This would result in more stably extracted pigments for compounds like pteridines and ommochromes, which can degrade fairly quickly in concentrated NaOH [24], but will extract considerably less melanin, thus making pteridine and ommochrome peaks most prominent. To determine whether these other pigments were present, extractions were followed by both absorbance spectrophotometry and thin layer chromatography (TLC). For TLC, samples were spotted on Macherey-Nagel (Düren, Germany) POLYGRAM CEL 400 cellulose plates and run using an 3% ammonium chloride solvent, dried, and viewed with both long and short wavelength UV light. If pteridine, ommochrome, or flavonoid pigments were present these would appear as fluorescent spots on the TLC plate.

Solubility and UV-Vis data for bumble bees have already been performed [15]. As a comparison to the velvet ants, the same extraction and spectrophotometry procedures were employed to extract red and black pigment from bumble bee pile. These analyses were performed on combined red pile from the second and third metasomal tergites of a single queen of *Bombus huntii*, avoiding any black or yellow hairs present on these segments, and on black abdominal pile from a queen of *Bombus impatiens*.

### Degradation analysis of melanin chemistry

#### Sampling

To determine the chemistry of the melanins we pooled colored tissue from as many specimens as possible for each tissue sample to ensure accurate melanin analysis. In an initial analysis, we examined orange-red setae from males and females of the bumble bee *Bombus huntii* (Fig 1; n = 200; 150 mg setae; specimens collected in Utah by J. Strange), a black setal sample taken from metasomal segments of queens and workers of both *Bombus impatiens* and *Bombus bimaculatus* Cresson (Fig 1; n = 75; 100 mg setae; collected from Koppert Biological Systems colonies or USA: Pennsylvania, State College), and an orange cuticle plus orange hair sample from the light orange version from females of the velvet ant, *Dasymutilla bioculata* (n = 2; 14 mg cuticle; color and collection data same as B6, Fig 1). In a subsequent analysis we analyzed melanin chemistry as part of three separate goals. First we analyzed velvet ants with different shades of orange-red, including cuticle + hairs from medium orange *Dasymutilla bioculata* (n = 2 dried/pinned females; 6 mg cuticle; color as in B5, Fig 1; USA: Arizona, Cochise Co., 21-Aug-1995, RR Snelling; Utah, Juab Co., nr. Mona, 18-ix-2003, RW Baumann and SM Clark) and dark orange *Dasymutilla bioculata* (n = 4 dried/pinned females; 15.8 mg cuticle; color as in B1 & B4, Fig 1; USA: Arizona, Santa Cruz Co., vii-viii-1980, RH Crandall), orange-red setae from the cow killer *Dasymutilla occidentalis* (n = 3 fresh-frozen females; 2.2 mg setae; DO Fig 1; USA: Alabama, Solon Dixon Rd.; 31.15, -86.70; vii-viii-2016; D. Owen), and black cuticle taken from legs, pleuron, sternites and posterior metasomal tergites of sampled *Dasymutilla bioculata*. Samples that included both colored hairs and the underlying cuticle were combined to attain a sufficient amount of material. While the color of the cuticle was the same for the dark orange *Dasymutilla*, the color of underlying cuticle in the light and medium orange *D*. *bioculata* was considerably darker than the lighter hairs. The primary cuticle was not included for *D*. *occidentalis* as this was completely black. Differences in tissue sampling in these specimens limits accurate comparison between different colored velvet ant samples, thus inferences in velvet ants are made more broadly.

Second, we analyzed black setae in queens of *Bombus impatiens* from two parts of the body, separating abdominal (metasomal) tergites 2+3 from tergites 4+5 (n = 11; 14.2 and 7.0 mg setae; from purchased Koppert colonies (USA) and USA: Pennsylvania, State College). Given that close relatives to this species (e.g., *Bombus ephippiatus* Say) can vary in whether the second and third segments are red or black [25] we sought to determine if the more anterior segments may contain differences in the amount of each melanin type from more posterior segments.

Finally, to analyze the nature of the red to black color shift in the different mimetic forms of *Bombus melanopygus*, we sampled from 2nd and 3rd metasomal segments orange-red hairs of queens of *Bombus melanopygus melanopygus* (n = 11, 14.2 mg setae) and black hairs from queens of *Bombus melanopygus edwardsii* (n = 9; 12.3 mg setae) sampled from multiple localities in the hybrid zone in southwest Oregon, USA. For all bumble bee samples the sampled segments are neighbored by the non-melanic yellow hairs and segmental coloration is discrete (not intermixed with other hair colors), therefore samples are largely pure in color and appeared to be monochrome when viewed under the microscope.

All dried and frozen specimens used for this study were obtained from material previously obtained for other purposes (dried/pinned mutillids from Utah State University, J. Wilson, J. Pitts; *B*. *huntii* from J. Strange, Utah State University; other bumble bees from H. Hines frozen collection, Pennsylvania State University). Specimens originally collected by the authors were obtained from University or other public lands not requiring permits or from purchased commercial colonies (e.g., *B*. *impatiens*, Koppert Biological Systems).

#### Chemical analysis

We used chemical degradation analysis of melanin to test for the presence of characteristic eumelanin and pheomelanin degradation products, including H_2_O_2_ oxidation products pyrrole-2,3,5-tricarboxylic acid (PTCA) and pyrrole-2,3-dicarboxylic acid (PDCA), and HI hydrolysis products 4-amino-3-hydroxyphenylalanine (4-AHP) and 4-amino-3-hydroxyphenylethylamine (4-AHPEA). PTCA is a eumelanin marker, especially abundant for 5,6-dihydroxyindole-2-carboxylic acid-derived, or DOPA-derived, eumelanin, while PDCA is a eumelanin marker proportionally more abundant for 5,6-dihydroxyindole-derived, or DA-derived, eumelanin. 4-AHP is a marker for the benzothiazine-unit in DOPA+Cys-derived pheomelanin and 4-AHPEA is a marker for the benzothiazine-unit in DA+Cys-derived pheomelanin.

All of the above tissue samples were homogenized with a Ten-Broeck glass homogenizer at a concentration of 5 mg tissue/mL water, thus standardizing by the amount of starting cuticular tissue. This involved using 0.5 mg of cuticle for all specimens, except for *D*. *occidentalis* for which 0.2 mg was used given limited available sample. For *D*. *occidentalis*, final metrics were adjusted to standardize by input weight (/mg). To examine degradation products of H_2_O_2_ oxidation, sample homogenate (100 μL) was placed in a 10 ml screw-capped conical test tube (large tube size enables vigorous vortexing), to which 375 μL 1 mol/L K_2_CO_3_ and 25 μL 30% H_2_O_2_ (final concentration: 1.5%) were added. The mixture was mixed vigorously at 25°C for 20 hr. The residual H_2_O_2_ was decomposed by adding 50 μL 10% Na_2_SO_3_ and the mixture was then acidified with 140 μL 6 mol/L HCl (caution was given to CO_2_ evolution). After vortexing, the reaction mixture was centrifuged at 4,000 g for 1 min, and an aliquot (80 μL) of the supernatant was directly injected into the HPLC system [26]. H_2_O_2_ oxidation products were analyzed with the HPLC system consisting of a JASCO 880-PU liquid chromatograph (JASCO Co., Tokyo, Japan), a Shiseido C_18_ column (Shiseido Capcell Pak MG; 4.6 x 250 mm; 5 μm particle size) and a JASCO UV detector. The mobile phase for analysis of PDCA and PTCA was 0.1 mol/L potassium phosphate buffer (pH 2.1): methanol, 99:1 (v/v). Analyses were performed at 45°C at a flow rate of 0.7 mL/min. Absorbance of the eluent was monitored at 269 nm.

For HI hydrolysis, sample homogenate (100 μL) was placed in a 10 ml screw-capped conical test tube, to which 20 μL 50% H_3_PO_2_ and 500 μL 57% HI were added. The tube was heated at 130°C for 20 hr, after which the mixture was cooled. An aliquot (100 μL) of each hydrolysate was transferred to a test tube and evaporated to dryness using a vacuum pump connected to a dry ice-cooled vacuum trap and two filter flasks containing NaOH pellets. The residue was dissolved in 200 μL 0.1 mol/L HCl. A 10 μL aliquot of each solution was analyzed on the HPLC system [27]. HI reductive hydrolysis products were analyzed with an HPLC system consisting of a JASCO 880-PU liquid chromatograph, a JASCO C_18_ column (JASCO Catecholpak; 4.6 x 150 mm; 7 μm particle size) and a Shiseido NanoSpace (3005 SI2) electrochemical detector. The mobile phase used for analysis of 4-AHP was 0.1 mol/L sodium citrate buffer, pH 3.0, containing 1 mmol/L sodium octanesulfonate and 0.1 mmol/L Na_2_EDTA:methanol, 98:2 (v/v). Analyses were performed at 35°C at a flow rate of 0.7 mL/min. The mobile phase used for analysis of 4-AHPEA was 0.1 mol/L sodium citrate buffer, pH 3.0, containing 1 mmol/L sodium octanesulfonate and 0.1 mmol/L Na_2_EDTA:methanol, 90:10 (v/v). Analyses were performed at 35°C at a flow rate of 0.7 mL/min. The electrochemical detector was set at +600 mV versus an Ag/AgCl reference electrode. PTCA, PDCA, 4-AHP, and 4-AHPEA used as standard samples for HPLC analyses were prepared using published protocols [21, 27–29]. An example of HPLC output used to measure the amount of each of these substances in one of our samples is provided in S1 Fig.

DA and DOPA-derived eumelanin and pheomelanin controls were analysed using the same methods as tissue samples above and were prepared as reported previously [21, 28–30]. 0.1 mg of each purified melanin were run through the reaction procedure (= 20% of the weight of tissue used). Overall concentrations of these melanins are likely to be higher than are to be expected in sampled cuticle, which is comprising mostly of proteins and chitin.

On each of the samples, we measured absorbance at 500 nm and 650 nm by using a Soluene-350 solubilization method [18]. The ratio of A500 and A650 reflects whether the sample is eumelanic or pheomelanic (0.25–0.33 for eumelanin; 0.10–0.15 for pheomelanin). To measure the absorbance of solubilized melanin, sample homogenate (100 μL) was placed in a 10 ml screw-capped conical test tube and 900 μL Soluene-350 (from PerkinElmer, Waltham, MA) was added. The tube received two rounds of vortexing and heating at 100°C (boiling water bath) for 15 min followed by cooling. After vortexing, the mixture was centrifuged at 4,000 g for 3–5 min, and the supernatant was analyzed for absorbance at 500 nm (A500) and 650 nm (A650). For a reference, a mixture of 100 μL water and 900 μL Soluene-350 was used after heating under the same conditions as for the samples [18].

## Results

### Solubility

**Fig 3:**
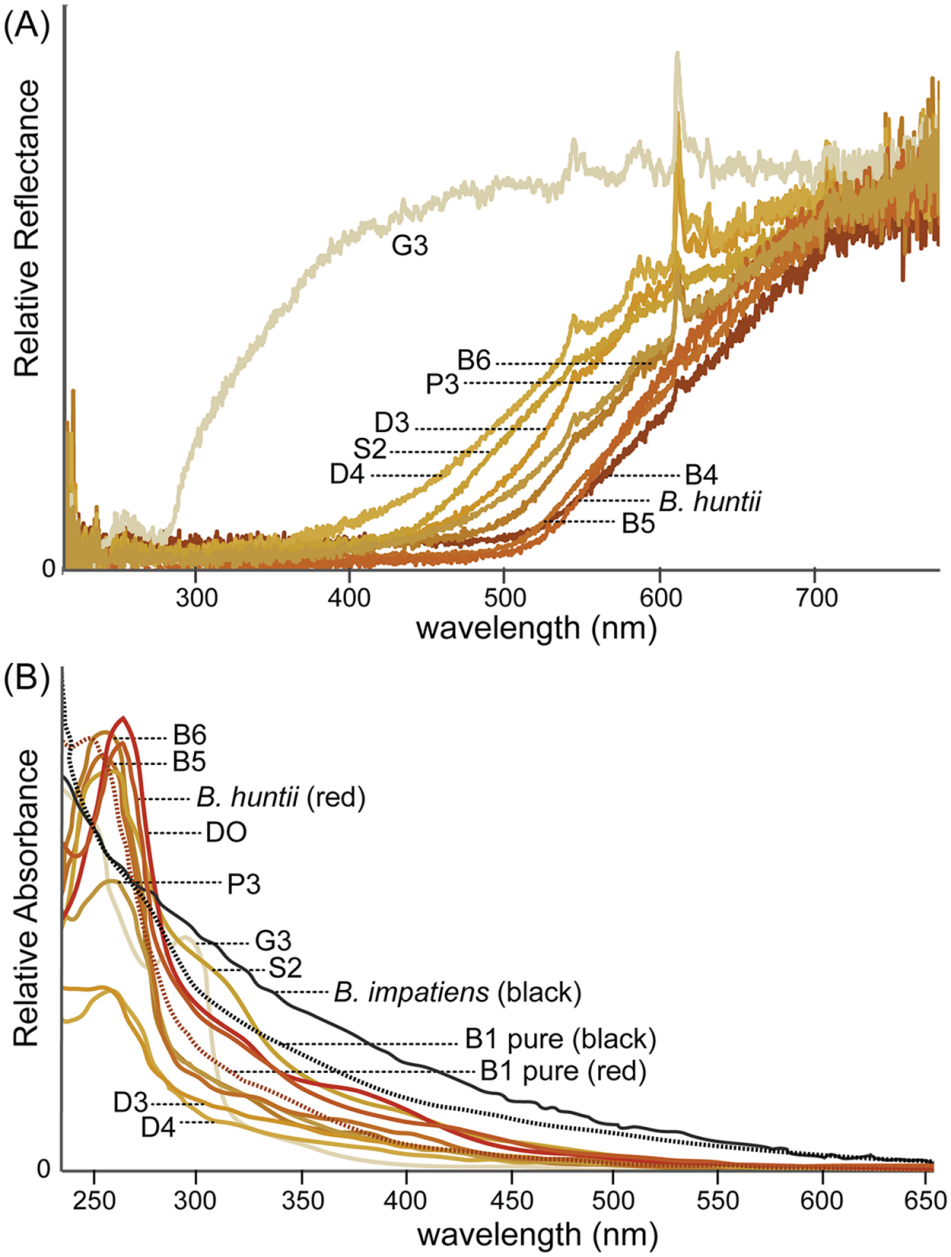
Reflectance (A) and absorbance (B) of specimens from Fig 1. Colors represent the color of the setal samples tested, measured from RGB data from photographs. Dashed lines in 2B indicate absorbance from melanin precipitate of mutillid B1. To enable better comparison of relative differences in shape of the absorbance and reflectance curves, reflectance curves have been standardized to a common value at 750 nm and absorbance curves have been adjusted to yield comparable levels between 225 and 275 nm, except in a few cases with especially low absorbances (D3,D4).

Tested velvet ants failed to yield a colored extract or absorbance spectra in mild acids or in acetone:hexane solvents suggesting that ommochromes or carotenoids do not comprise their colors (Table 1; S1 Supporting Information). 0.01 M Na_2_CO_3_ extracts yielded a faint color in some cases and very little absorbance in the visible range in the spectrophotometer (Table 1; S1 Supporting Information). Substantial color was leached for all samples (except white *D*. *gloriosa*) in 0.1 M pH 13 NaOH, and yielded spectra with substantial absorbance in visible wavelengths (Table 1 and Fig 3B). These results match patterns obtained from red and black bumble bee hair setae and are suggestive of the pigments from both velvet ants and bumble bees being melanins. That these are melanins is also supported by the precipitation of the pigment in both black and red *Dasymutilla bioculata* setal samples (specimen B1) upon addition of 0.1 M pH 1 HCl to the pH 13 NaOH extracts.

**Table 1:** Results of solubility tests, spectrophotometry, and TLC on sampled velvet ants and bumble bees.

Species	ID	UV fluor.	pH10 0.01M Na_2_CO_3_	pH3 MeOH	Acetone: Hexane (5:1)	pH13 0.1M NaOH	Conclusion
***Dasymutilla satanas***	D3	----	Weak color, melanin spectrum	No color or spectrum	No Color	Color, melanin spectrum	Melanin
***Dasymutilla occidentalis***	DO	No*	Weak color, melanin spectrum*	No color or spectrum	No Color	Color, melanin spectrum	Melanin
***Bombus huntii***		----	Weak color, melanin spectrum	No color or spectrum	No Color	Color, melanin spectrum	Melanin
***Bombus impatiens***		----	Weak color, melanin spectrum	No color or spectrum	No Color	Color, melanin spectrum	Melanin
***Dasymutilla gloriosa***	G3	No	No color, weak melanin spectrum	No color or spectrum	----	No color, A peak around 300 nm	?
***Dasymutilla scitula***	S2	No	Weak color, melanin spectrum	----	----	Color, melanin spectrum + other peaks?	Melanin
***Psorthaspis portiae***	P3	No	Weak color, melanin spectrum	----	----	Color, melanin spectrum + other peaks?	Melanin
***Dasymutilla satanas***	D4	----	----	----	----	Color, melanin spectrum	Melanin
***Dasymutilla bioculata***	B6	----	----	----	----	Color, melanin spectrum	Melanin
***Dasymutilla bioculata***	B5	----	----	----	----	Color, melanin spectrum	Melanin
***Dasymutilla bioculata***	B1	----	----	----	----	Color, melanin spectrum	Melanin

ID = ID of the specimen used for most extractions, referred to in Fig 1 and the text.

Different specimens of the same color were used for *D*. *gloriosa*, *D*. *scitula*, and *P*. *portiae* Na_2_CO_3_ extractions (S1 Supporting Information).

---- = not tested.

fluor = fluorescence in TLC.

* see S1 Supporting Information for further information.

### UV-Vis reflectance, absorbance, and spectral ratios

Reflectance data of all velvet ant and spider wasp specimens showed a pattern expected for melanin, with a reflectance slope that steadily increases in the visible range (Fig 3A). As would be expected for their color, the lighter the hue from yellow to red the more reflectance towards shorter wavelengths, with more red hues reflecting proportionally more in the longer wavelengths. *D*. *gloriosa* on the other hand reflects nearly fully across the visible range, which would result in the white color. Black color (not shown) has no reflectance across all wavelengths. Bumble bee reflectance spectra match to those of similarly colored mutillids (Fig 2A).

Absorbance spectra show similar but inversed patterns (Fig 3B). For both red and black colored individuals of both bumble bees and velvet ants/spider wasps, absorbance occurs in a steadily descending slope from UV towards visible wavelengths. These patterns are those expected for melanins and do not match any of the alternative pigments which would have defined peaks within the visible range rather than a descending slope. Similarly, the colors match patterns of absorbance typically seen in pheomelanins vs. eumelanins, with the red melanins absorbing proportionally more light in the shorter wavelengths than black. Pigment precipitate of both black and orange-red setae from the same specimens (dashed line, Fig 3B) redissolved in 0.1 M NaOH demonstrate that the pigment is indeed what has precipitated and the resulting slopes show this relative shift between red and black absorbance spectra nicely. While black for both velvet ants and bumble bees has proportionately more absorbance in the 400+ nm visible absorbance range than the red samples, the absorbance pattern of the velvet ant black are closer to the pheomelanin pattern than the bumble bees. *D*. *gloriosa* (white) provides a baseline for expected non-absorbance of white specimens.

Some of the velvet ant and spider wasp specimens yielded extra shoulders in absorbance in the 280 - 400nm absorbance range (namely P3, DO, G3, and S2) that could be indicative of other pigments in addition to melanin. However, when we extracted individuals of each of these species with 0.01 M Na_2_CO_3_, which should extract most pigments yielding peaks such as this (pteridines, ommochromes, flavonoids), we did not obtain any peaks beyond the melanin slope and samples of both NaOH and Na_2_CO_3_ extractions run on thin layer chromatography did not yield fluorescent spots. Either these pigments occur in such small amounts that they not detectable with the methods used or they may be products of harsh NaOH treatment of melanins that are not fluorescent. *D*. *gloriosa* yielded a much more substantial peak around 295 nm which should be pursued further regarding its chemical nature.

The 650/500nm ratios of the UV-Vis spectra from purified extracts of specimens used for degradation analysis (Table 2) suggested all orange-red setae and cuticle samples of both bumble bees and mutillids are pheomelanins and all black samples eumelanin. The black hairs in bumble bees yielded a ratio that matches eumelanin (between 0.305 and 0.331 across bumble bee samples; 0.338 for Dopa-melanin, 0.280 for Dopamine-melanin). The black cuticular samples of the velvet ant *D*. *bioculata*, however, was intermediate between the eumelanin and pheomelanins in its ratios (0.231 for the velvet ant black; 0.132 for Dopa pheomelanin and 0.142 for Dopamine pheomelanin), suggesting it may be comprised of considerable amount of pheomelanin in addition to eumelanin. Bumble bee and velvet ant red-orange samples were similar in their ratios and closer to the ratios of pheomelanin, ranging between 0.168 and 0.205 across all samples. These data match observations from absorbance spectra (Fig 3B).

**Table 2:** Quantitative HPLC values and relative ratios from H_2_O_2_ oxidation, HI hydrolysis, and Soluene-350 analysis of samples relative to melanin controls.

		Soluene-350 (/mg)		H2O2 Oxidat. (ng/mg)		HI Hydrolysis (ng/mg)		Ratios				
	Sample	A500	A650	PTCA	PDCA	4-AHP	4-AHPEA	A650/A500	PDCA/PTCA	4-AHP /PTCA	4-AHPEA/PDCA	4-AHPEA/4-AHP
	*B*. *huntii* orange	0.200	0.037	8.49	25.1	9.58	1094	0.185	2.96	1.13	43.6	114.2
	*B*. *melanopygus* orange	0.204	0.040	11.9	26.0	53.7	1870	0.196	2.19	4.53	72.1	34.9
	*B*. *melanopygus* black	0.996	0.330	302	404	28.9	530	0.331	1.34	0.10	1.31	18.3
	*B*. *impatiens/bimac*. black	0.861	0.263	252	378	96.1	228	0.305	1.50	0.38	0.60	2.4
	*B*. *impatiens* black T2+T3	0.750	0.232	210	338	7.4	260	0.309	1.61	0.04	0.77	35.3
	*B*. *impatiens* black T4+	0.898	0.296	301	429	14.4	372	0.330	1.42	0.05	0.87	25.8
	*D*. *bioculata* light orange	0.119	0.020	18.6	49.7	5.74	350	0.168	2.67	0.31	7.04	61.0
	*D*. *bioculata* med orange	0.072	0.014	5.9	22.3	5.5	198	0.194	3.80	0.94	8.90	36.0
	*D*. *bioculata* dark orange	0.074	0.014	3.4	16.4	7.4	140	0.189	4.88	2.19	8.56	19.0
	*D*. *occidentalis* red	0.220	0.045	8.4	13.1	22.9	782	0.205	1.55	2.73	59.9	34.1
	*D*. *bioculata* black	0.268	0.062	55.9	91.2	3.7	283	0.231	1.63	0.07	3.10	76.4
	Dopa-melanin	8.81	2.98	4790	5780	1110	ND	0.338	1.21	0.23	1/inf	1/inf
	DA-melanin	5.96	1.67	2790	4750	ND	1200	0.280	1.70	1/inf	0.25	inf
	Dopa+Cys-melanin	4.79	0.63	4230	857	130000	867	0.132	0.20	30.7	1.01	0.01
	DA+Cys-melanin	2.46	0.35	17.7	1940	ND	112000	0.142	110	1/inf	57.7	inf

Colors reflect color of original sample taken from photographic RGB data.

For ease of interpretation, we highlighted cells based on similarity to melanin controls, with lighter shades indicating values in between melanin types.

inf = infinity.

### Melanin degradation-product analysis

Hydrogen peroxide oxidation yields high amounts of chemical degradation products PTCA and PDCA in eumelanin samples derived from both DOPA and dopamine, although proportionally more PDCA is generated by dopamine-derived eumelanins. The combined yield of these products is lower in pheomelanins, but DOPA+cys-derived pheomelanins produce proportionally more PTCA and dopamine+cys-derived pheomelanins mostly PDCA, making amounts and ratios of these products meaningful for determining the types and derivatization of melanins. Results from the degradation analysis, standardized to weight of starting tissue, revealed much higher and more similar levels of these chemical degradation products in all black bumble bee samples (Table 2). Ratios match more closely to the 1.7 PDCA/PTCA ratio of dopamine eumelanin than the 1.2 ratio of DOPA eumelanin although some values, such as *B*. *melanopygus* black hairs, approach that of DOPA eumelanin (1.34). The black mutillid cuticle sample yielded overall amounts of PDCA and PTCA intermediate between black bumble bee and red setal samples, but ratios are similar to dopamine eumelanin. Much lower levels of both of these degradation products occur in the orange-red samples of both mutillids and bumble bees. Ratios between PDCA and PTCA support considerably more PDCA in these red samples, suggesting that these bees are utilizing mostly dopamine-derived pheomelanins. In *D*. *occidentalis*, which is more of a red color, the amounts of both are lower but ratios are also considerably lower, perhaps suggesting proportionally more DOPA pheomelanin may be present.

HI hydrolysis of melanin yields the degradation products 4-AHP and 4-AHPEA. 4-AHP is only yielded in DOPA-derived melanins and 4-AHPEA in dopamine-derived melanin. However, the levels of these are much higher in pheomelanins than in eumelanins, making these derivatives good indicators as well of both types and derivatization of these melanins. Ratios between these suggest that none of these samples exclusively contain DOPA or dopamine melanins and suggest that dopamine-derived melanins are the predominant melanins across all samples. Levels of 4-AHPEA are highest in bumble bee orange-red hairs and the *D*. *occidentalis* red hairs and are much higher than 4-AHP levels in these individuals, supporting dopamine-derived pheomelanin as the primary contributor to red coloration in both lineages. Although levels of the other orange velvet ants are fairly low, the ratio of the dopamine-derived pheomelanin signature (4-AHPEA) to the dopamine-derived eumelanin signature (PDCA) is much higher than the black samples. This suggests that pheomelanins predominate but that lower levels are present in orange hairs of *D*. *bioculata* per unit tissue sampled than are seen in other orange-red samples. Considerable variation in ratios of 4-AHP to 4-AHPEA levels across samples suggest that while dopamine-derived melanins are always more common, the relative amounts of DOPA vs. dopamine-derived melanins differ. Results from more anterior black segments in *B*. *impatiens* do not differ much from more posterior segments, thus likely have similar melanic composition.

The high levels of both H_2_O_2_ and HI degradative markers in *B*. *melanopygus* black hairs but still levels at ~⅓ what is seen in the red form for HI hydrolysis, in addition to the elevated 4-AHPEA/PDCA ratio in the black form when compared to control eumelanin, suggests that black form individuals likely contain both eumelanin and pheomelanin, whereas red form individuals contain primarily pheomelanin. Similarly, the intermediate levels found in this ratio in the black cuticle of the velvet ant suggests this too is likely a combination of pheomelanin and eumelanin.

## Discussion

Our data provide support for the black and orange-red variation in both bumble bees and velvet ant mimicry systems being a result of melanic shifts, thus implicating targeted modification of the melanin pathway in their rampant mimetic color changes. The reddish aposematic pigments in bumble bees and velvet ants are determined to be generated by high concentrations of pheomelanins. Pheomelanins were thought not to occur in insects until recently. This study adds to a few other recent examples [22,23] in supporting the use of pheomelanins to attain yellow-reddish colors and cuticular sclerotization in insects. One of the other two insect examples of pheomelanins was from a parasitic wasp [23], also is in the order Hymenoptera. It is fairly common for Hymenopterans to have shades of reddish-brown cuticle in place of black and therefore pheomelanin-eumelanin variation may be a fairly common and more ancestral condition for this lineage.

Black samples in the velvet ants and the bumble bees contained some pheomelanin, thus it is likely that modification of the relative amounts of different melanins deposited into insect cuticle could explain color in these insects. In the bumble bee *Bombus melanopygus*, convergence onto the black Pacific coastal versus the red Rocky mountain color pattern (Fig 1) involves a switch between mostly eumelanin combined with some pheomelanin (black form), to mostly pheomelanin (red form). Thus, identifying the genetics underlying this trait will rely on isolating the enzymes whose expression enables the transition between eumelanin and pheomelanin.

Our study found that eumelanin and pheomelanin in these insects were mostly dopamine derived, although in all cases some pheomelanin and eumelanin was DOPA-derived. Furthermore, samples varied in the relative contribution of each precursor. Cys-dopamine derived pheomelanins were also more predominant in the *Sphingonotus* grasshopper [22] and in general insects contain more dopamine than DOPA eumelanins (e.g., [31]). Given the established melanin pathway, ample *dopa decarboxylase* in insects would enable conversion of most DOPA to dopamine. Pheomelanins could then be produced by facilitating cysteine addition to this dominant precursor. Leading genes implicated in the light to dark shifts in dopamine-derived melanin products are *tan*, *ebony*, and *aaNAT*. *tan* converts dopamine to dopamine-derived eumelanin, *ebony* counteracts the effect of *tan*, converting dopamine instead to tan-colored NBAD sclerotins, and *aaNAT* drives dopamine toward the clear NADA sclerotins. Pheomelanin synthesis likely represents an additional branch to this pathway [22].

The use of dopamine-derived pheomelanin is in contrast to the normal pattern for vertebrates. Most vertebrate pheomelanins involve the addition of cysteine to DOPA. Dopamine-derived pheomelanins in vertebrates involve a neurotoxic reactive oxidative intermediate 5-S-Cys-dopamine. Furthermore, in vertebrates, pheomelanin synthesis occurs through interactions with the antioxidant glutathione which is consumed by pheomelanin production [32,33]. Thus, red plumage or hairs has been considered to potentially be teratogenic, predisposing their beholders to more mutagenic effects when exposed to high oxidative stress [32, 34–36].

If insect melanins are primarily dopamine derived and if the pathways are similar to vertebrates, one might conclude that insects with reddish color may be more predisposed to negative impacts of oxidative stress. This argument, however, is contradictory to the distribution patterns of red coloration in bumble bees. Red is more common in higher altitudes in New World bumble bees [14,37]. Part of this has to do with convergence onto the higher altitude Rocky Mountain red mimicry pattern as opposed to the lower altitude coastal and east temperate black patterns. However, if red was indeed more susceptible to oxidative damage, these bees would remain at a disadvantage in high altitudes where oxidative stress would be higher. In velvet ants the red patterns occur in both eastern and western North American habitats at various altitudes, the darker colors are more common in tropical or subtropical regions, and the lighter yellow and white colors are more common in the deserts. The patterns of darkest and lightest coloration in these velvet ants match what is observed in bumble bees, where darker colors are also highest in the tropics and lighter colors in the most exposed desert habitats [12]. Most likely, a combination of mimetic frequency-dependent selection on historical patterns, thermoregulatory properties (explains light desert colors), and immune advantages of darker colors (explains darker colors in the tropics, i.e., Gloger’s rule) are involved in their coloration. However, a role of the toxicity of the red pigments in these insects is worth further investigation, especially in light of these insects using this red coloration as a signal of their own toxicity.

This study highlights how much there is to be discovered in insect pigmentation. The velvet ants and bumble bees are some of our most charismatic and evolutionarily intriguing insects. Diving into their pigmentation has revealed that they both harbor pigments that once were thought not to occur in insects, through the pheomelanins and their potential diversity, but also the discovery of a novel yellow pigment in bumble bees. This raises the question of how many more pigment types are yet to be discovered across the megadiverse insects.

## Supporting information

S1 Fig HPLC analyses after the chemical degradation of *B*. *melanopygus* orange-form setal hairs. (A) H_2_O_2_ oxidation. #1: PDCA (26.0 ng/mg), #2: PTCA (11.9 ng/mg). Retention time: #1 (14.9 min), #2 (18.9 min). Attenuation: 8. (B) HI hydrolysis. #3: 4-AHP (53.7 ng/mg). Retention time: #3 (32.2 min). Attenuation: 32 (C) HI hydrolysis. #4: 4-AHPEA (1870 ng/mg). Retention time: #4 (9.6 min). Attenuation: 512.
(PDF)

S1 Supporting Information Additional absorbance spectra of samples run in solubility tests. (A) *Bombus impatiens* black hairs extracted in Na_2_CO_3_. (B) *Bombus huntii* orange and yellow hairs extracted in Na_2_CO_3_. (C) *Dasymutilla occidentalis* extracted in Na_2_CO_3_. (D) *Dasymutilla scitula* S1 extracted in Na_2_CO_3_. (E) *Psorthaspis portiae* P1 extracted in Na_2_CO_3_. (F) *Dasymutilla satanas* extracted in Na_2_CO_3_. (G) *Dasymutilla gloriosa* G1 extracted in Na_2_CO_3_. (H) *Dasymutilla gloriosa* G1 extracted in pH3 MeOH. These spectra were used to make spectral inferences presented in Table 1, not including the spectra obtained from NaOH, which are presented in Fig 3. Each spectrum is labelled by species and the extraction buffer used to obtain the extract. Only samples that yielded absorbance are shown. A few Na_2_CO_3_ used different specimens from those used in the NaOH extraction. Identification code and photos of these specimens are shown. Spectra were run on a Nanodrop spectrophotometer and include either saved image files from the Nanodrop or files plotted from exported data. *Bombus huntii* orange hairs (B) yielded faint peaks that match the pteridine-like spectrum of the yellow bumble bee pigment, suggesting the orange hairs may contain some of the yellow pigment within them. The yellow spectrum from setae from the same bee sampled in the same relative amount of setae and using the same protocol as the orange hairs is compared to the orange-setal spectrum to visualize the extent of the difference in signal. *Dasymutilla occidentalis* yielded some peaks in the UV range in the Na_2_CO_3_ extraction in addition to the melanin spectrum. TLC was run on this sample and yielded no fluorescent spots, thus it is unlikely this is a pteridine, flavonoid, or ommochrome.
(DOCX)

## References

[1] Turner JRG. Adaptation and evolution in *Heliconius*: a defense of NeoDarwinism. Annu Rev Ecol Syst 1981;12: 99–121.

[2] Sheppard PM, Turner JRG, Brown KS, Benson WW, Singer MC. Genetics and the evolution of Muellerian mimicry in *Heliconius* butterflies. Philos Trans R Soc Lond B Biol Sci 1985;308: 433–610.

[3] Symula R, Schulte R, Summers K. Molecular phylogenetic evidence for a mimetic radiation in Peruvian poison frogs supports a Müllerian mimicry hypothesis. Proc R Soc Lond B Biol Sci 2001; 268: 2415–2421. 10.1098/rspb.2001.1812 PMC1088895 11747559

[4] Sanders KL, Malhotra A, Thorpe RS. Evidence for a Müllerian mimetic radiation in Asian pitvipers. Proc R Soc Lond B Biol Sci 2006; 273: 1135–1141. 10.1098/rspb.2005.3418 PMC1560257 16600892

[5] Marek PE, Bond JE. A Müllerian mimicry ring in Appalachian millipedes. Proc Natl Acad Sci U S A 2009;106: 9755–9760. doi: 10.1073/pnas.0810408106 19487663 10.1073/pnas.0810408106 PMC2700981

[6] Starr CK. A simple pain scale for field comparison of hymenopteran stings. J Entomol Sci 1985; 20: 225–231.

[7] Schmidt JO, Blum MS, Overal WL. Hemolytic activities of stinging insect venoms. Arch Insect Biochem Physiol 1985;1: 155–160.

[8] Schmidt J. Hymenopteran venoms: striving toward the ultimate defense against vertebrates In: Insect defenses: adaptive mechanisms and strategies of prey and predators. Albany: State University of New York Press; 1990 pp. 387–419.

[9] Wilson JS, Williams KA, Forister ML, von Dohlen CD, Pitts JP. Repeated evolution in overlapping mimicry rings among North American velvet ants. Nat Commun 2012; 3: 1272 doi: 10.1038/ncomms2275 23232402 10.1038/ncomms2275

[10] Wilson JS, Jahner JP, Forister ML, Sheehan ES, Williams KA, Pitts JP. North American velvet ants form one of the world’s largest known Müllerian mimicry complexes. Curr Biol 2015;25:R704–R706. doi: 10.1016/j.cub.2015.06.053 26294178 10.1016/j.cub.2015.06.053

[11] Rodriguez J, Pitts JP, von Dohlen CD, Wilson JS. Müllerian mimicry as a result of codivergence between velvet ants and spider wasps. PLoS One 2014;9: e112942 doi: 10.1371/journal.pone.0112942 25396424 10.1371/journal.pone.0112942 PMC4232588

[12] Williams P. The distribution of bumblebee colour patterns worldwide: possible significance for thermoregulation, crypsis, and warning mimicry. Biol J Linn Soc Lond 2007;92, 97–118.

[13] Rapti Z, Duennes MA, Cameron SA. Defining the colour pattern phenotype in bumble bees (*Bombus*): a new model for evo devo. Biol J Linn Soc Lond 2014;113: 384–404.

[14] Franklin HJ. The Bombidae of the new world. Trans Amer Entomol Soc 1912;38, 177–486.

[15] Hines HM. Bumble bees through the ages: historical biogeography and the evolution of color diversity. PhD thesis, University of Illinois Urbana-Champaign, Urbana, IL. 2008.

[16] Ito S, Wakamatsu K. Quantitative analysis of eumelanin and pheomelanin in humans, mice, and other animals: a comparative review. Pigment Cell Res 2003;16: 523–531. 12950732 10.1034/j.1600-0749.2003.00072.x

[17] Galván I, Jorge A, Ito K, Tabuchi K, Solano F, Wakamatsu K. Raman spectroscopy as a non-invasive technique for the quantification of melanins in feathers and hairs. Pigment Cell Melanoma Res 2013;26: 917–923, doi: 10.1111/pcmr.12140 23890020 10.1111/pcmr.12140

[18] Ozeki H, Ito S, Wakamatsu K, Thody AJ. Spectrophotometric characterization of eumelanin and pheomelanin in hair. Pigment Cell Res 1996;9: 265–270. 9014213 10.1111/j.1600-0749.1996.tb00116.x

[19] Ito S, Wakamatsu K. Chemical degradation of melanins: application to identification of dopamine-melanin. Pigment Cell Res 1998;11: 120–126. 9585251 10.1111/j.1600-0749.1998.tb00721.x

[20] Wakamatsu K, Ito S. Advanced chemical methods in melanin determination. Pigment Cell Res 2002;15: 174–183. 12028581 10.1034/j.1600-0749.2002.02017.x

[21] Ito S, Fujita K, Yoshioka M, Sienko D, Nagatsu T. Identification of 5-S- and 2-S-cysteinyldopamine and 5-S-glutathionyldopamine formed from dopamine by high-performance liquid chromatography with electrochemical detection. J Chromatogr B Biomed Appl 1986;375: 134–140.

[22] Galván I, Jorge A, Edelaar P, Wakamatsu K. Insects synthesize pheomelanin. Pigment Cell Melanoma Res 2015;28: 599–602. doi: 10.1111/pcmr.12397 26176957 10.1111/pcmr.12397

[23] García AJ, Polidori C, Nieves-Aldrey JL. Pheomelanin in the secondary sexual characters of male parasitoid wasps (Hymenoptera: Pteromalidae). Arthropod Struct Dev 2016; 45: 311–319. doi: 10.1016/j.asd.2016.05.001 27224206 10.1016/j.asd.2016.05.001

[24] Kayser H. Pigments. Comprehensive Insect Physiology, Biochemistry and Pharmacology. 1985; 10: 367–415.

[25] Duennes MA, Lozier JD, Hines HM, Cameron SA. Geographical patterns of genetic divergence in the widespread Mesoamerican bumble bee *Bombus ephippiatus* (Hymenoptera: Apidae). Mol Phylogenet Evol 2012;64: 219–231. doi: 10.1016/j.ympev.2012.03.018 22521295 10.1016/j.ympev.2012.03.018

[26] Ito S, Nakanishi Y, Valenzuela RK, Brilliant MH, Kolbe L, Wakamatsu K. Usefulness of alkaline hydrogen peroxide oxidation to analyze eumelanin and pheomelanin in various tissue samples: application to chemical analysis of human hair melanins. Pigment Cell Melanoma Res 2011;24: 605–613. doi: 10.1111/j.1755-148X.2011.00864.x 21535429 10.1111/j.1755-148X.2011.00864.x

[27] Wakamatsu K, Ito S, Rees JL. The usefulness of 4-amino-3-hydroxyphenylalanine as a specific marker of pheomelanin. Pigment Cell Res 2002;15: 225–232. 12028587 10.1034/j.1600-0749.2002.02009.x

[28] D’Ischia M, Wakamatsu K, Napolitano A, Briganti S, Garcia-Borron J-C, Kovacs D, et al Melanins and melanogenesis: methods, standards, protocols. Pigment Cell Melanoma Res 2013;26: 616–633. doi: 10.1111/pcmr.12121 23710556 10.1111/pcmr.12121

[29] Wakamatsu K, Ohtara K, Ito S. Chemical analysis of late stages of pheomelanogenesis: conversion of dihydrobenzothiazine to a benzothiazole structure. Pigment Cell Melanoma Res 2009;22: 474–486. doi: 10.1111/j.1755-148X.2009.00580.x 19493317 10.1111/j.1755-148X.2009.00580.x

[30] Wakamatsu K, Fujikawa K, Zucca FA, Zecca L, Ito S. The structure of neuromelanin as studied by chemical degradative methods. J Neurochem 2003;86: 1015–1023. 12887698 10.1046/j.1471-4159.2003.01917.x

[31] Sugumaran M. Comparative biochemistry of eumelanogenesis and the protective roles of phenoloxidase and melanin in insects. Pigment Cell Res 2002;15: 2–9. 11837452 10.1034/j.1600-0749.2002.00056.x

[32] Galván I, Bonisoli‐Alquati A, Jenkinson S, Ghanem G, Wakamatsu K, Mousseau TA, et al Chronic exposure to low‐dose radiation at Chernobyl favours adaptation to oxidative stress in birds. Funct Ecol 2014; 28: 1387–1403.

[33] Galván I, Solano F. Melanin chemistry and the ecology of stress. Physiol Biochem Zool 2015;88: 352–355. doi: 10.1086/680362 25860833 10.1086/680362

[34] Galván I, Timothy AM, Møller AP. Bird population declines due to radiation exposure at Chernobyl are stronger in species with pheomelanin-based coloration. Oecologia 2011;165: 827–835. doi: 10.1007/s00442-010-1860-5 21136083 10.1007/s00442-010-1860-5

[35] Roulin A, Almasi B, Meichtry‐Stier KS, Jenni L. Eumelanin‐and pheomelanin-based colour advertise resistance to oxidative stress in opposite ways. J Evol Biol 2011;24: 2241–2247. doi: 10.1111/j.1420-9101.2011.02353.x 21745253 10.1111/j.1420-9101.2011.02353.x

[36] Napolitano A, Panzella L, Monfrecola G, d'Ischia M. Pheomelanin‐induced oxidative stress: bright and dark chemistry bridging red hair phenotype and melanoma. Pigment Cell Melanoma Res 2014;27: 721–733. doi: 10.1111/pcmr.12262 24814217 10.1111/pcmr.12262

[37] Koch JB. Biogeography, Population Genetics, and Community Structure of North American Bumble Bees. PhD thesis, Utah State University, Utah. 2015.

