# Supplementary figures and images for: Melanic variation underlies aposematic color variation in two hymenopteran mimicry systems
Source: PLoS One. 2017 Jul 28;12(7):e0182135. doi: 10.1371/journal.pone.0182135 (PMC5533327; doi:10.1371/journal.pone.0182135)

**A**

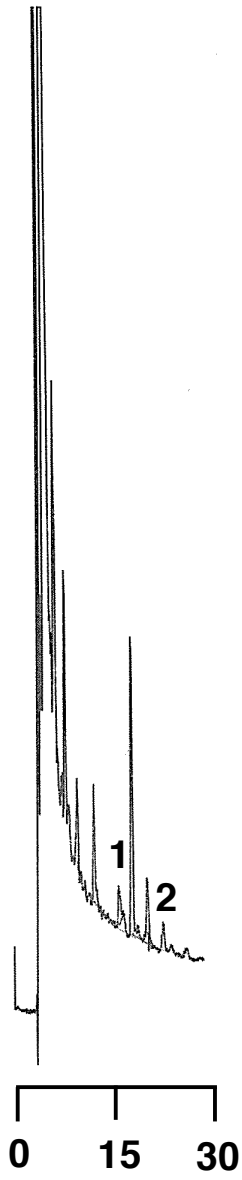

**B**

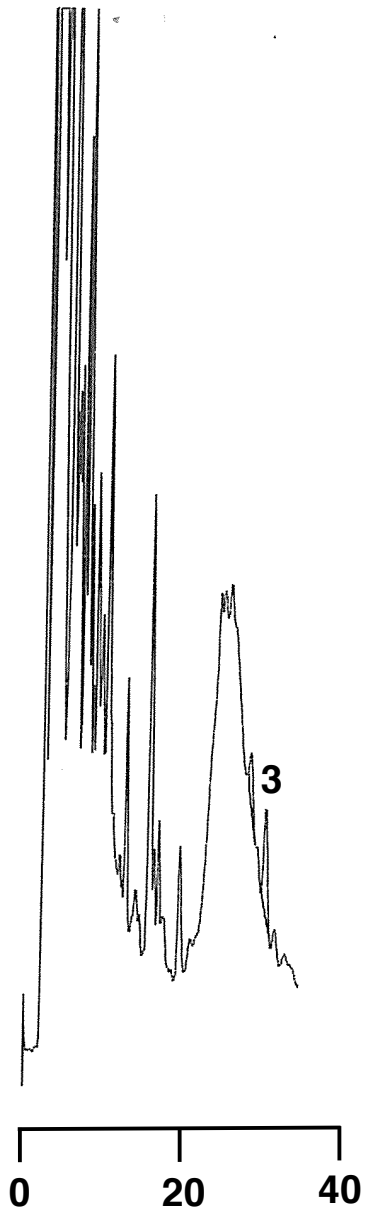

**C**

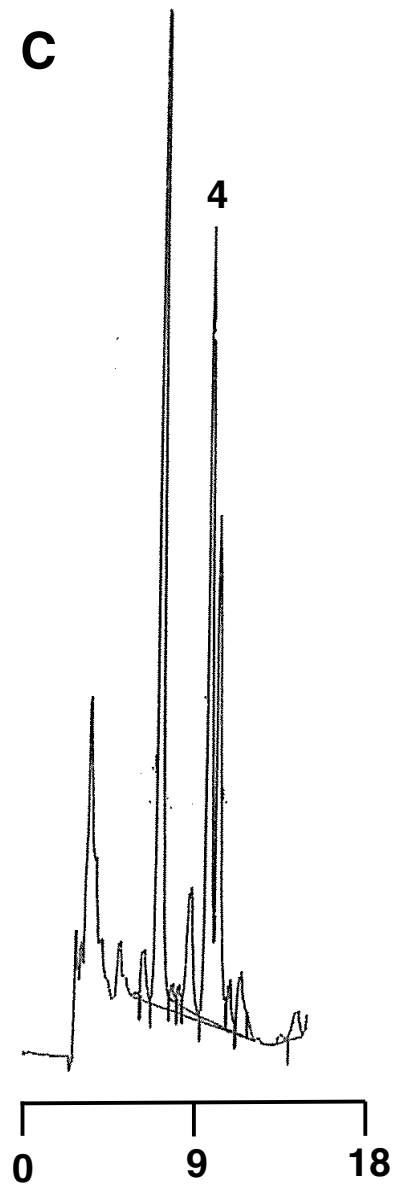

**Retention time (min.)**

Supplement: S1 Fig — (A) H2O2 oxidation. #1: PDCA (26.0 ng/mg), #2: PTCA (11.9 ng/mg). Retention time: #1 (14.9 min), #2 (18.9 min). Attenuation: 8. (B) HI hydrolysis. #3: 4-AHP (53.7 ng/mg). Retention time: #3 (32.2 min). Attenuation: 32 (C) HI hydrolysis. #4: 4-AHPEA (1870 ng/mg). Retention time: #4 (9.6 min). Attenuation: 512. (PDF) [file pone.0182135.s001.pdf]
