# Supplementary material for: Melanic variation underlies aposematic color variation in two hymenopteran mimicry systems
Source: PLoS One. 2017 Jul 28;12(7):e0182135. doi: 10.1371/journal.pone.0182135 (PMC5533327; doi:10.1371/journal.pone.0182135)
Supplement: S1 Supporting Information — (A) Bombus impatiens black hairs extracted in Na2CO3. (B) Bombus huntii orange and yellow hairs extracted in Na2CO3. (C) Dasymutilla occidentalis extracted in Na2CO3. (D) Dasymutilla scitula S1 extracted in Na2CO3. (E) Psorthaspis portiae P1 extracted in Na2CO3. (F) Dasymutilla satanas extracted in Na2CO3. (G) Dasymutilla gloriosa G1 extracted in Na2CO3. (H) Dasymutilla gloriosa G1 extracted in pH3 MeOH. These spectra were used to make spectral inferences presented in Table 1, not including the spectra obtained from NaOH, which are presented in Fig 3. Each spectrum is labelled by species and the extraction buffer used to obtain the extract. Only samples that yielded absorbance are shown. A few Na2CO3 used different specimens from those used in the NaOH extraction. Identification code and photos of these specimens are shown. Spectra were run on a Nanodrop spectrophotometer and include either saved image files from the Nanodrop or files plotted from exported data. Bombus huntii orange hairs (B) yielded faint peaks that match the pteridine-like spectrum of the yellow bumble bee pigment, suggesting the orange hairs may contain some of the yellow pigment within them. The yellow spectrum from setae from the same bee sampled in the same relative amount of setae and using the same protocol as the orange hairs is compared to the orange-setal spectrum to visualize the extent of the difference in signal. Dasymutilla occidentalis yielded some peaks in the UV range in the Na2CO3 extraction in addition to the melanin spectrum. TLC was run on this sample and yielded no fluorescent spots, thus it is unlikely this is a pteridine, flavonoid, or ommochrome. (DOCX) [file pone.0182135.s002.docx]

***
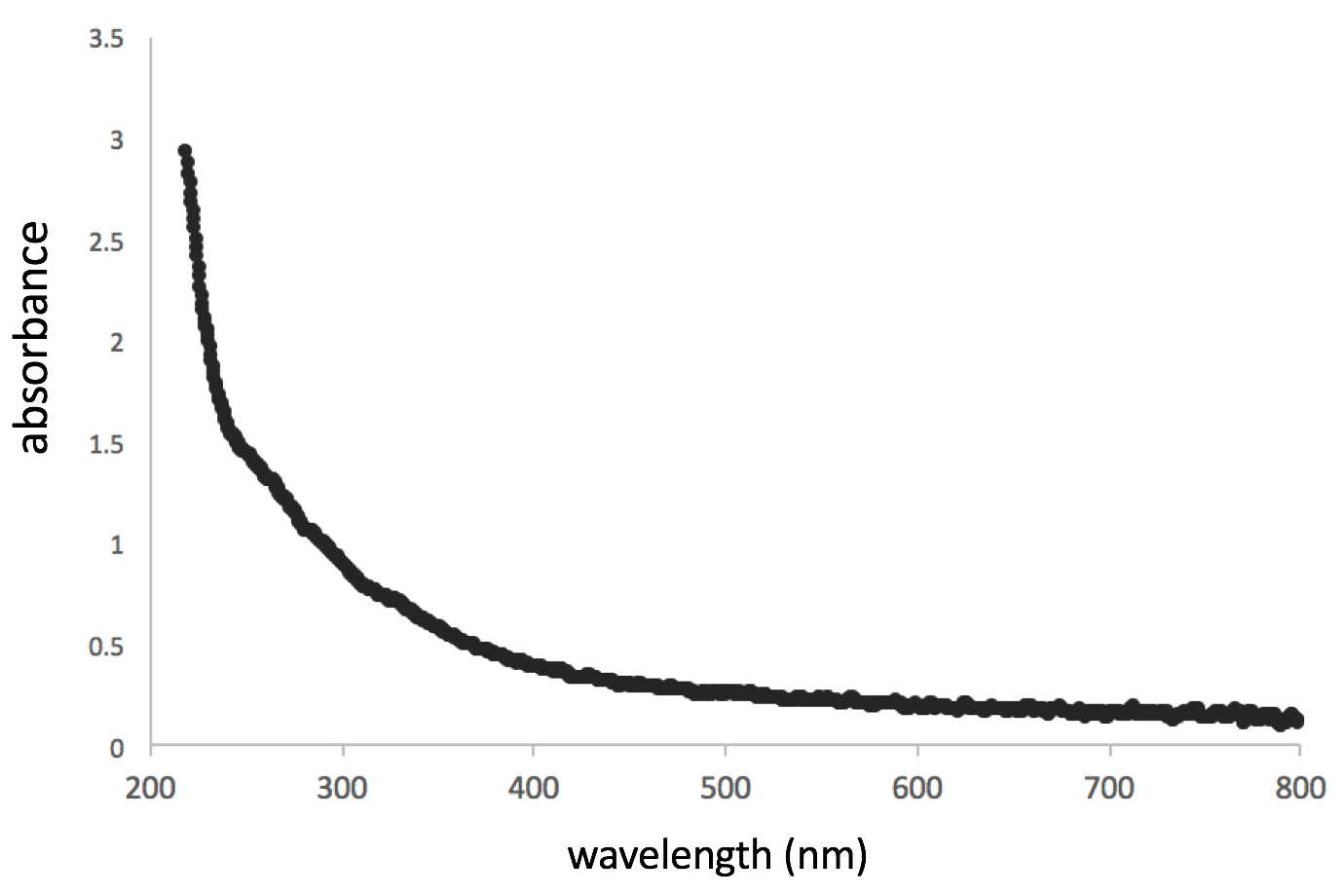
***

1. *Bombus impatiens* black hairs extracted in Na_2_CO_3_

***
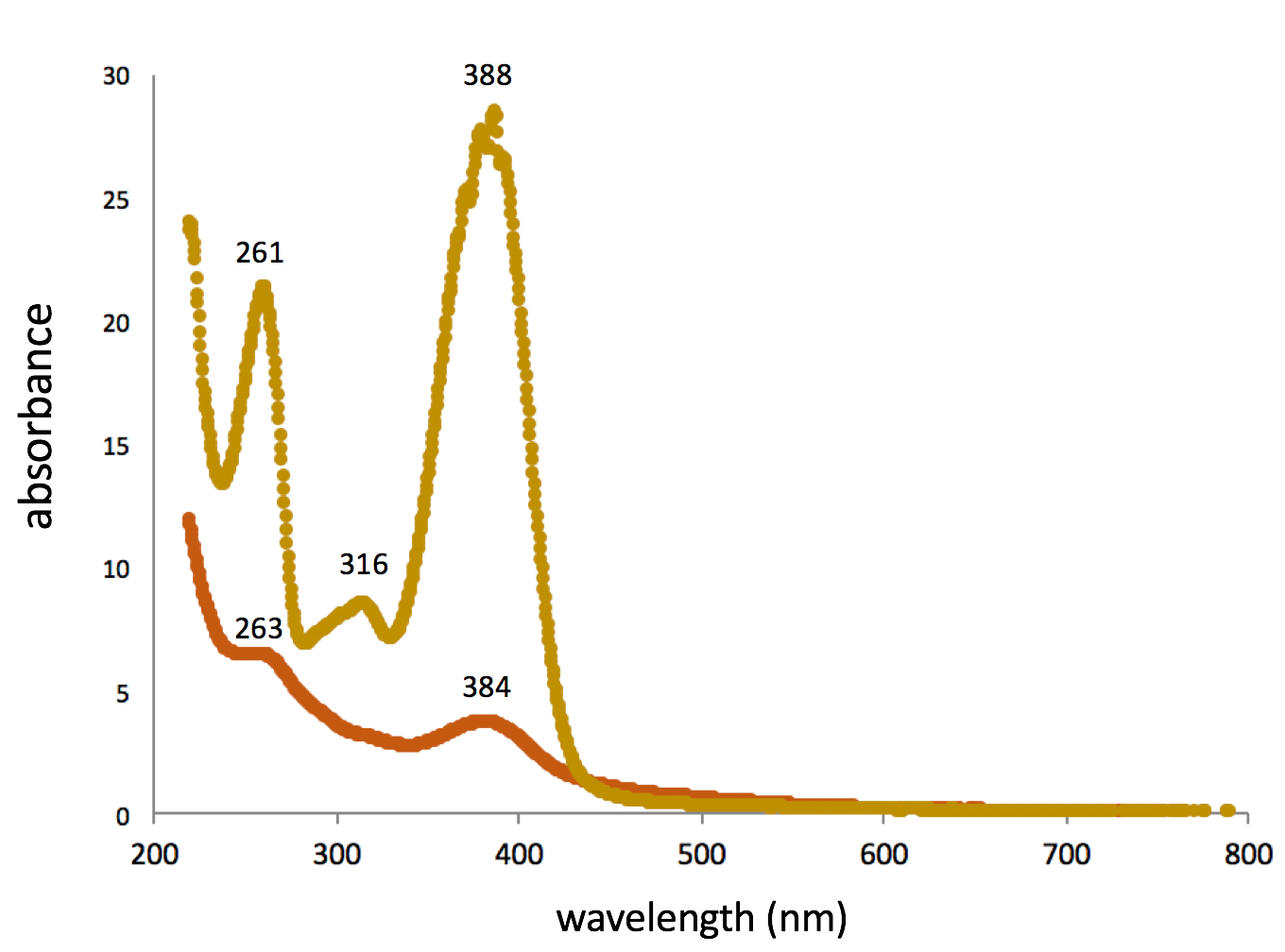
***

1. *Bombus huntii* orange and yellow hairs extracted in Na_2_CO_3_

***
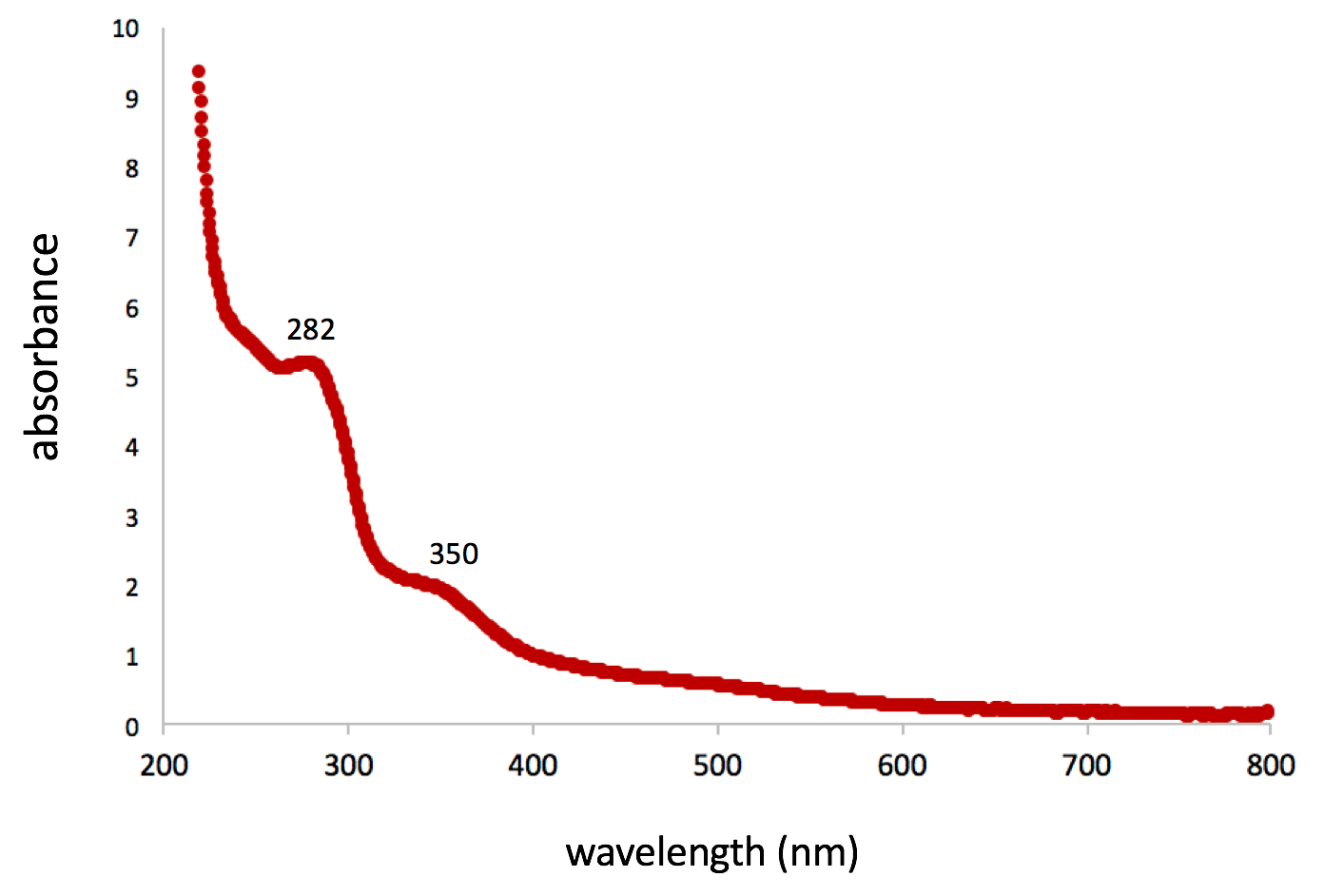
***

1. *Dasymutilla occidentalis* extracted in Na_2_CO_3_


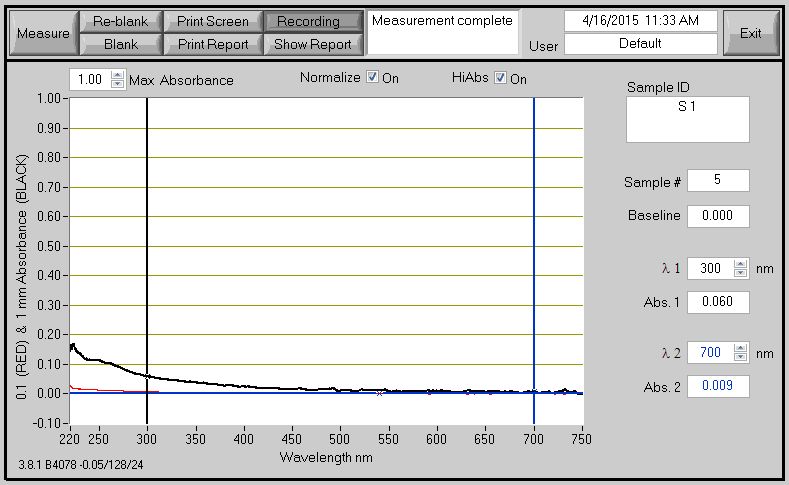
 ***
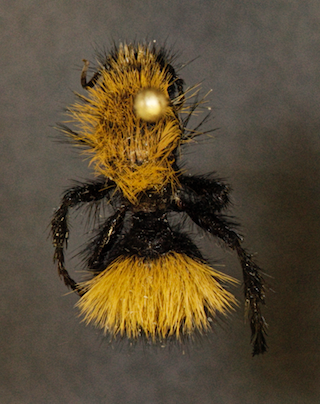
***

(D) *Dasymutilla scitula* S1 extracted in Na_2_CO_3_ **S1**


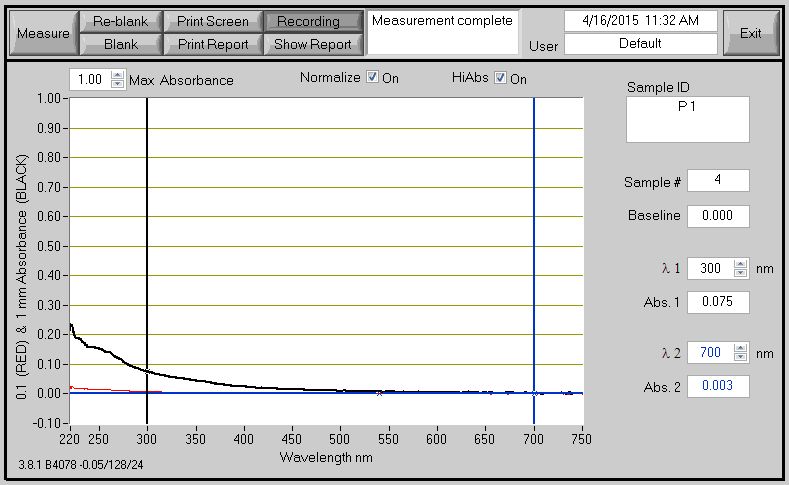

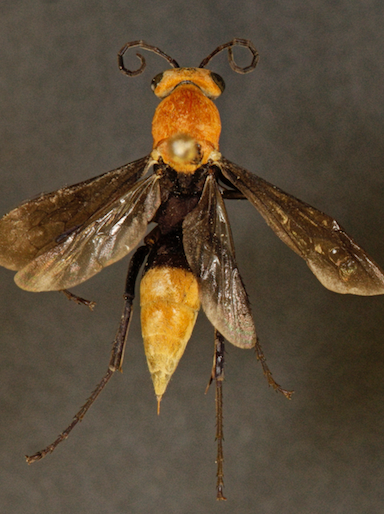


(E) *Psorthaspis portiae* P1 extracted in Na_2_CO_3_ **P1**


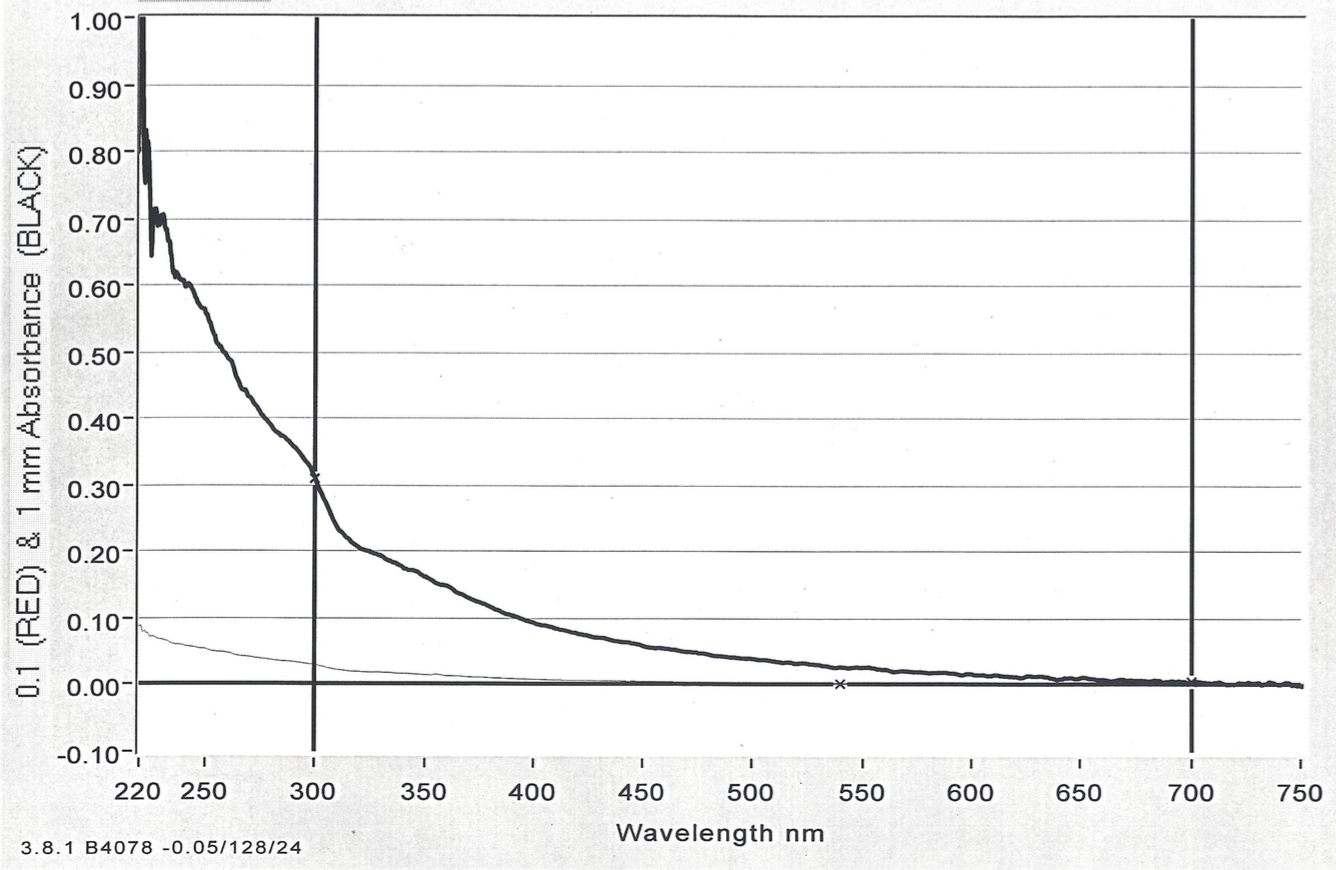


(F) *Dasymutilla satanas* extracted in Na_2_CO_3_


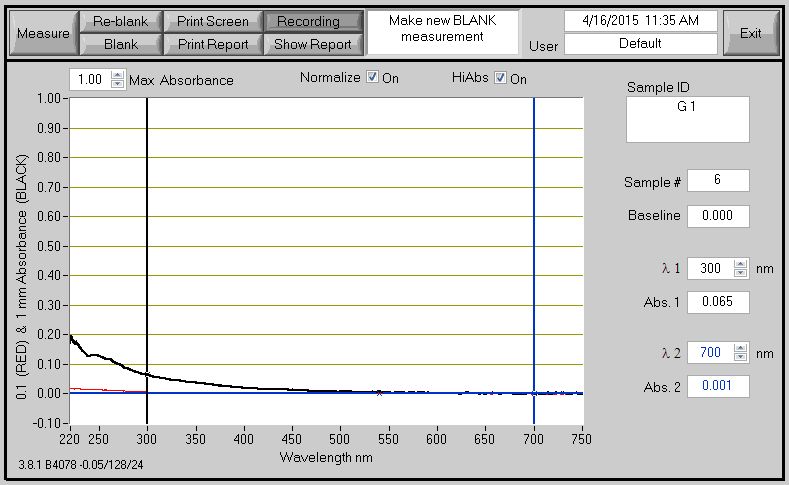

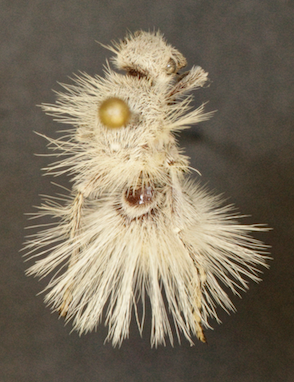


(G) *Dasymutilla gloriosa* G1 extracted in Na_2_CO_3_  **G1**


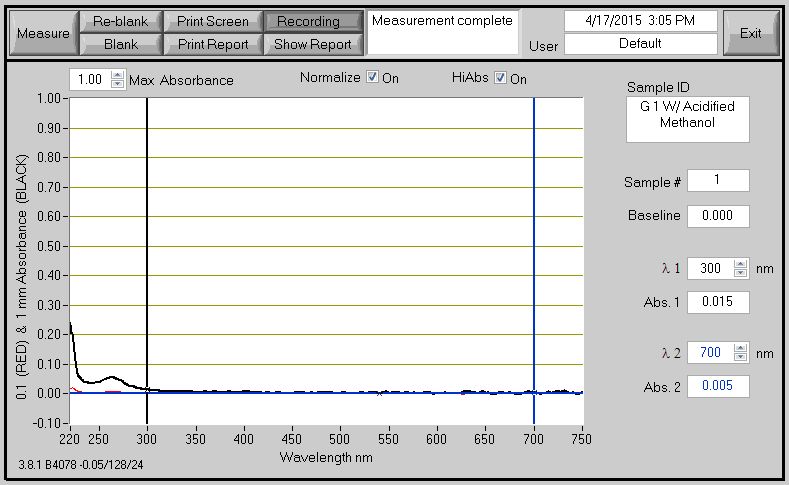


(H) *Dasymutilla gloriosa* G1 extracted in pH3 MeOH
